# Nanocellulose: The Ultimate Green Aqueous Dispersant for Nanomaterials

**DOI:** 10.3390/polym16121664

**Published:** 2024-06-12

**Authors:** Víctor Calvo, Carlos Martínez-Barón, Laura Fuentes, Wolfgang K. Maser, Ana M. Benito, José M. González-Domínguez

**Affiliations:** Instituto de Carboquímica (ICB-CSIC), C/Miguel Luesma Castán 4, 50018 Zaragoza, Spain; cmartinez@icb.csic.es (C.M.-B.); fuentesvarelaura@gmail.com (L.F.); wmaser@icb.csic.es (W.K.M.); abenito@icb.csic.es (A.M.B.)

**Keywords:** cellulose, dispersions, nanocrystals, nanofibers, nanomaterials

## Abstract

Nanocellulose, a nanoscale derivative from renewable biomass sources, possesses remarkable colloidal properties in water, mechanical strength, and biocompatibility. It emerges as a promising bio-based dispersing agent for various nanomaterials in water. This mini-review explores the interaction between cellulose nanomaterials (nanocrystals or nanofibers) and water, elucidating how this may enable their potential as an eco-friendly dispersing agent. We explore the potential of nanocellulose derived from top-down processes, nanocrystals, and nanofibers for dispersing carbon nanomaterials, semiconducting oxide nanoparticles, and other nanomaterials in water. We also highlight its advantages over traditional methods by not only effectively dispersing those nanomaterials but also potentially eliminating the need for further chemical treatments or supporting stabilizers. This not only preserves the exceptional properties of nanomaterials in aqueous dispersion, but may even lead to the emergence of novel hybrid functionalities. Overall, this mini-review underscores the remarkable versatility of nanocellulose as a green dispersing agent for a variety of nanomaterials, inspiring further research to expand its potential to other nanomaterials and applications.

## 1. Introduction

Cellulose, as the most abundant biopolymer on earth, is a key raw material in the search for sustainability on an industrial scale. It is a linear polysaccharide composed of polymerized D-glucose molecules linked by β-1,4-glucosidic bonds [1], forming a rigid network due to its rich hydroxyl-based surface composition [1,2]. Found as the main structural component in plants, like cotton or hemp [3], cellulose is typically found within a matrix alongside other biopolymers (hemicellulose, lignin, proteins, etc.), organic substances, and trace elements, requiring extraction to isolate pure cellulose [4]. Cellulose exhibits different crystallographic structures (allomorphs) based on chain orientation. Amongst the six different types, type I, with its chains arranged in parallel, is predominant in nature, while the usually manmade type II displays its chains in an anti-parallel manner [1].

**Figure 1:**
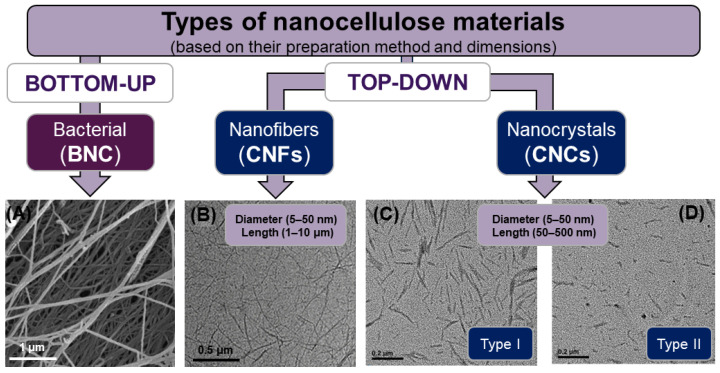
Classification of different types of nanocellulose materials according to their preparation method and dimensions. Electron microscopy images of each nanocellulose material adapted from different publications: (**A**) SEM of BNC adapted from reference [7] (Copyright © 2020, Springer), (**B**) TEM of CNFs adapted from reference [8] (Copyright © 2018, American Chemical Society), and (**C**) and (**D**) TEM of both allomorphs CNCs adapted from reference [9] (Copyright © 2019, American Chemical Society).

In recent years, there has been a growing interest within the scientific community regarding nanomaterials derived from cellulose. Termed broadly as nanocellulose, these materials have at least one dimension in the nanoscale range, between 1 and 100 nm, while retaining the chemical structure of cellulose [3]. Nanocellulose combines the inherent properties of cellulose, like biocompatibility or mechanical resistance, with unique characteristics arising from their nanoscale dimensions, such as their enhanced water stability, unlocking their potential for a myriad of applications. The general classification includes three types of nanocellulose materials based on their preparation method and dimensions (Figure 1): bacterial nanocellulose (BNC), cellulose nanocrystals (CNCs), and cellulose nanofibers (CNFs). BNC (Figure 1A) is prepared via a bottom-up process, wherein specific aerobic bacteria produce nanofibers that end up entangled in the shape of a dense hydrogel at the interface between the culture medium and air [5]. In particular, BNC is formed directly at the nanoscale from a carbon source, such as glucose or fructose. It offers a high water retention capacity, purity, and crystallinity, expanding its versatility for applications in environmental remediation, healthcare, food packaging, and surface coatings, among others [5,6,7].

Conversely, CNFs (Figure 1B) and CNCs (Figure 1C) are both synthesized through top-down methodologies, with variations in dimensions attributed to the synthesis conditions [3]. CNFs possess a flexible, elongated morphology with a nanoscale diameter and micron-scale length [10]. The production methods for CNFs from plant cellulose are classified according to the applied synthesis protocols: (i) mechanical treatments, such as high-pressure homogenization or ultrasonication; (ii) soft chemical cleavage, usually through oxidative and/or acid-catalyzed reactions; and (iii) biological or enzymatic degradation [10]. CNFs can serve as a reinforcement constituent in composites, enhancing mechanical properties, or as a structural component in 3D-printed hydrogel scaffolds [11,12].

Although CNCs are prepared in a similar top-down process as CNFs, they are notably shorter, exhibiting only tens to hundreds of nanometers in length (Figure 1C,D) [13]. CNCs also reveal a stiff rod-like structure, contributing to enhanced mechanical resistance and superior water stability [13]. CNCs possess exceptional mechanical strength, biocompatibility, low toxicity, and environmental sustainability, making them attractive candidates for a wide range of applications, such as the stabilization of Pickering emulsions, the reinforcement of polymers, or biomedical applications. In general, the production of CNCs from cellulose is based on chemical processes, such as acid hydrolysis or TEMPO oxidation, or enzymatic treatments [3,14,15]. Provided that almost every existent cellulose in nature exhibits the type I allomorph, an additional step is normally required to prepare type II CNCs, which is typically accomplished through mercerization (wet treatment with concentrated NaOH), TEMPO oxidation, or recrystallization processes [9,16]. However, these traditional processes raise concerns about sustainability and can significantly alter the surface chemistry of CNCs [14,17]. This is why the one-pot acid hydrolysis method for directly obtaining type II CNCs is attracting significant attention [9,18]. This method involves the adjustment of synthesis conditions to favor the swelling of cellulose chains before hydrolysis, leading to the targeted cleavage of amorphous regions and the rearrangement into type II CNCs [9,18]. As shown in Figure 1C,D, the allomorph significantly impacts the size and shape of the nanocrystals and also the mass yield, with a 25% for type I and a 40% for type II [18]. Furthermore, it influences various other properties, such as the enzymatic degradation rate [19] and the ability to interact with other materials, which are important for applications like the stabilization of Pickering emulsions [6].

This mini-review aims to analyze the potential of using two types of cellulose nanomaterials, CNCs and CNFs, for the dispersion of other nanomaterials in aqueous media. First, we proceed to evaluate the colloidal properties of nanocellulose materials derived from top-down processes. By analyzing relevant publications, we aim to showcase the immense potential of nanocellulose as a key player in developing eco-friendly and green solutions for nanotechnological applications.

## 2. Nanocellulose-Water Interactions and Their Implications for Its Aqueous Colloidal Properties

**Figure 2:**
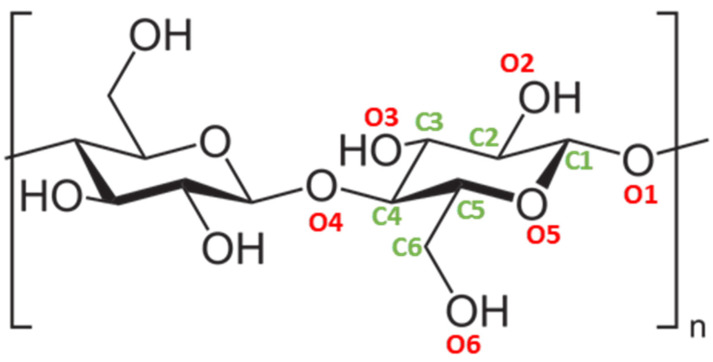
The repeating unit of cellulose, also known as cellobiose, composed of 2 units of β-1,4-linked glucose.

Understanding how nanocellulose interacts with water is crucial before evaluating its potential for processing other nanomaterials in aqueous environments. These interactions follow the basis of those from bulk cellulose, dictating the aqueous behavior of their nanoscale analogs. The amphiphilic nature of cellulose, governed by its chemical composition and crystalline structure, drives its strong affinity for water. The hydroxyl-rich surface, able to form hydrogen bonds with water molecules, is the main driving force behind the strong hydrophilic character of nanocellulose. However, the behavior of each individual hydroxyl group in the cellobiose units when interacting with water molecules varies depending on their location within the cellulose backbone (Figure 2). O2 and O6 are the most hydrophilic ones, acting directly as hydrogen-bond donors to water. O3 is also kind of hydrophobic, but it can only accept hydrogen bonds from water because it donates its own proton intramolecularly to the adjacent hydroxyl group (O5), which imparts a hydrophobic character to O5 [20,21]. Therefore, O3, O2, and O6 fall somewhere between the most and least water-attracting groups.

Paradoxically, hydrogen bonds are also the reason for cellulose’s insolubility in water, given the strong attachment experienced by adjacent polymer chains (particularly in the more compact crystallite regions) that cannot be disrupted by spontaneous water solvation. Thus, cellulose is a fascinating polymer with great avidity for water retention but never truly dissolves in it. This interaction depends on the specific crystal structure exposed to the cellulose surface. Most crystal faces, like (110) and (010), are quite attracted to water (highly hydrophilic), while others, like (100), are less. Notably, the (200) plane in cellulose I_β_ is even considered water-repelling (hydrophobic) [20,22]. As a general rule, an overall higher degree of crystallinity impedes the access of water molecules towards OH groups [23]. Moreover, there is also an effect of the water uptake with respect to polymorphism. Type II cellulose is reported to adsorb more water than type I allomorph, but the reason is not fully understood. Most likely, it relates to the fact that type II has more exposed OH groups on the surface or to its lower overall crystallinity degree, which is concomitant to the type I to type II interconversion process [23].

For nanocellulose prepared via acid hydrolysis with sulfuric acid, water plays a dual role as reaction medium and reagent [18]. During the hydrolysis process, amorphous chain cleavage and the concomitant random sulphation of the resulting CNCs, or CNFs, lead to water adsorption while still suspended in the aqueous medium. Nanocellulose exhibits remarkable water uptake ability due to its high surface area-to-volume ratio, allowing for easier access to hydroxyl groups compared to its bulk counterpart [23]. However, as a distinct and unique feature, nanocellulose can form stable aqueous colloidal suspensions. The negative surface charges incorporated by sulfate ester groups upon reaction with H_2_SO_4_ contribute to this stability through electrostatic repulsion between nanocellulose particles [18,24].

The most suitable parameter used to assess the colloidal stability of a nanocellulose suspension is its ζ-potential. In general, a suspension is considered stable when its absolute ζ-potential value surpasses 30 mV [18,21,23]. Being highly sensitive to surface charge, pH, ionic strength, and certain surface modifications [23], well defining the experimental conditions for the measurement of ζ-potential is of critical importance. Notably, achieving a stable water-based nanocellulose colloid critically depends on the processing techniques used: sonication and centrifugation. On the one hand, a high energy input in sonication is indispensable for the dispersion of solid powders or for the dilution of pre-existing aqueous colloids. Ultrasounds are preferred due to their efficient cavitation forces, which promote the formation of hydrodynamic envelopes around nanocellulose particles [23]. On the other hand, after sonication, centrifugation comes into play to stabilize nanocellulose in suspension. While most studies agree that centrifugation is the common method for stabilizing nanocellulose suspensions, only Benselfelt et al. [24] explicitly claimed and emphasized its importance for the formation of stable nanocellulose dispersions to eliminate larger aggregates.

Indeed, the outstanding water-holding capacity, high aspect ratio, structural rigidity, and anisotropy of nanocellulose materials, grant them unique gelation properties. Gel-like behavior has been observed even in dilute suspensions, with CNCs and CNFs exhibiting gelation at mass concentrations as low as 8–10 wt%, and 2–3 wt%, respectively [20,23]. For a deeper dive into the topic of the gelation properties of nanocellulose, not being the central pillar within this mini-review, please refer to specific reviews like the one by Curvello et al. [25]. Interestingly, nanocellulose can go beyond mere physical gelation and form self-standing hydrogels upon hydrothermal treatments that promote crosslinking via sulfate ester groups, forming a dense network that retains water [26,27].

**Figure 3:**
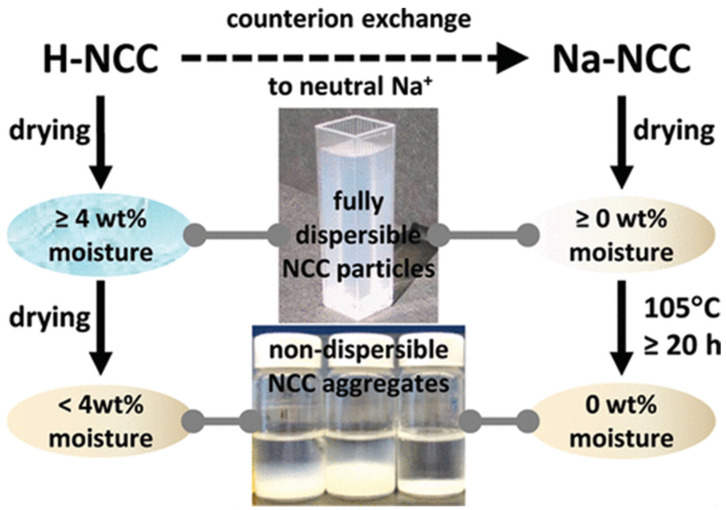
Dispersibility roadmap regarding moisture content and ionic compensation in nanocellulose dried powders. Reproduced with permission from reference [28]. Copyright © 2012, American Chemical Society.

Another important factor influencing colloidal stability is the moisture content of nanocellulose in its solid form. Several studies have reported a moisture threshold (around 4 wt%) below which nanocellulose becomes impossible to redisperse in water (Figure 3) [28,29]. Thus, maintaining a good moisture content in the powder is essential to ensuring it will still easily disperse in water and remain usable over time. Falling below the minimum moisture threshold leads to “hornification”, an irreversible phenomenon where nanocellulose loses its ability to be resuspended in liquids [30]. This fact, already observed for bulk cellulose, although to a lesser extent, given the high active surface area of nanocellulose, is attributed to complex crystallizations. These formations lead to hydrogen-bond cross-linking that permanently obstructs the access of water molecules towards the hydroxyl groups [23]. Thus, careful control of drying steps is crucial to preserving this residual moisture content (at least 4 wt%) and maintaining processability. Such a unique structural dynamic of nanocellulose with moisture has been exploited by some authors to create high-sensitivity strain sensors [31].

Beyond moisture content, the re-dispersibility of dried nanocellulose powders can also be tuned by ionic forces. Forming a neutral sodium-based salt counterion onto the surface of nanocellulose (achieved, for instance, by compensating acidic sites with sodium cations from NaCl or NaOH) can hinder the irreversible aggregation of hydrogen bonds, facilitating redispersion in water (Figure 3) [30]. To this end, it is common practice to add small amounts of a soluble sodium salt to the aqueous nanocellulose colloids before drying, ensuring stable colloidal behavior upon resuspension. In a similar way, the addition of organic adjuvants, such as glycerol or polyglycols, contributes to overcoming problems related to hornification [30,32].

Significant research is ongoing to develop practical methods for dewatering or drying nanocellulose while preserving its exceptional properties. This challenge stems from knowledge acquired in the classical cellulose industry. Sinquefield and co-workers excellently reviewed this topic [20]. Its keypoints can be summarized as follows: In the first place, drying cellulose or nanocellulose samples is important for practical reasons related to storage and shipping issues, where large volumes of water are not desirable. However, current drying techniques still challenge the ability to fully preserve the nanoscale features of nanocellulose, hindering its commercialization potential. The high-water affinity of cellulose, due to the presence of multiple hydroxyl groups, makes complete dehydration difficult. For nanocellulose, the existence of sulfate ester groups further complicates this process, since it requires overcoming even stronger molecular interactions. There are several experimental drying methods, including evaporation (with or without vacuum), heat-based drying, freeze drying (also known as lyophilization), supercritical CO_2_ drying, and spray drying. While each one represents its own pros and cons [20,23,32], they all rely on the appropriate choice of the solvent, ionic composition, and additives used in the nanocellulose colloidal system. Only nanocellulose powders with sufficiently minimal self-aggregation will effectively redisperse, maintaining the same properties as the parent colloid. In general terms, Sinquefield et al. [20] affirm that no single dewatering or drying method yet fulfills all the ideal characteristics: non-destructive, cost-effective, low energy consumption, scalable, and environmentally friendly. These authors suggest that a deeper understanding of the thermodynamics of water retention by nanocellulose could pave the way for the ultimate drying technology for nanocellulose [20].

## 3. Carbon Nanomaterials Aqueous Dispersions via Nanocellulose

**Figure 4:**
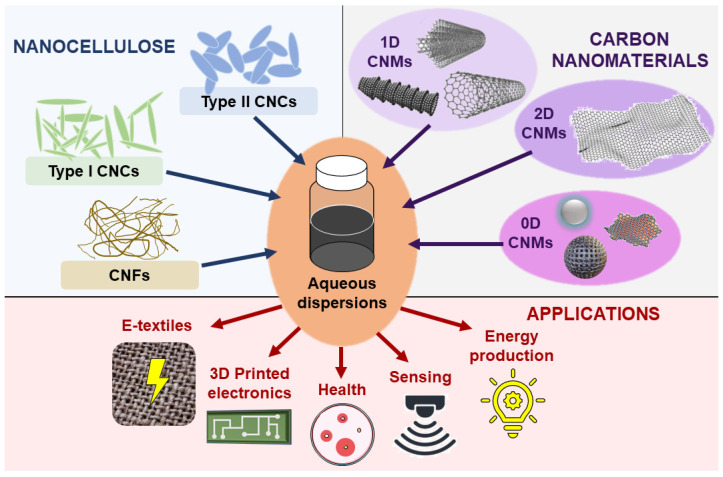
General scheme of the combination of nanocellulose materials and CNMs in dispersions and their potential applications.

Carbon nanomaterials (CNMs) represent a group of materials with at least one dimension in the nanoscale, between 1 and 100 nm, and with carbon as their main element [33]. Different examples are single-walled or multiwalled carbon nanotubes (SWCNTs or MWCNTs), graphene, and carbon dots (CDs) [33]. The carbon sp^2^ hybridization and their dimensions provide CNMs with outstanding properties, such as high electrical conductivity, high surface area, or mechanical resistance [34]. CNMs hold significant potential for applications across a variety of fields, including electronics, sensors, materials science, energy storage, and biomedicine [33]. CNMs are often classified based on the number of dimensions on the nanoscale: zero-dimensional (0D), one-dimensional (1D), and two-dimensional (2D) [33]. However, despite their remarkable attributes, CNMs often encounter challenges in transferring their properties to useful commercial products due to their inherent hydrophobicity and tendency to agglomerate in liquid dispersion [33,35]. To mitigate this shortage, strategies such as surface chemistry modifications and the use of organic solvents or surfactants are commonly employed [36,37]. However, it is important to note that these modifications can potentially alter the properties of nanomaterials and raise concerns regarding their environmental impact. Recently, nanocellulose materials have emerged as a green alternative to typical surfactants, such as sodium dodecylbenzene sulfonate (SDBS), Triton^®^ or Pluronic^®^, standing themselves as a greener choice for the dispersion of CNMs in water (Figure 4, Table 1) [9,38,39,40].

1D CNMs, such as SWCNTs, MWCNTs, and carbon nanofibers, are cylindrical structures that exhibit exceptional mechanical strength, high electrical conductivity, and thermal stability [33,35]. A pioneering study by Olivier et al. in 2011 demonstrated the possibility of using CNCs to disperse SWCNTs in water [39]. They proposed utilizing type I CNCs, prepared by H_2_SO_4_ hydrolysis, to introduce sulfate groups that would facilitate the stabilization of the SWCNTs [39,41]. Through layer-by-layer assembly with a polyelectrolyte, they successfully fabricated conductive thin films while preserving the electronic properties of SWCNTs, suggesting potential applications in sensing [39,41]. Years later, the same research team, led by Olivier Chauvet, extended the use of type I CNCs for dispersing other SWCNTs and MWCNTs [42]. Their findings revealed that a higher CNCs concentration is required to disperse MWCNTs compared to SWCNTs and emphasize the importance of optimizing the sonication time and other parameters to ensure stable colloids [42]. Furthermore, they developed ultra-low-density CNTs-reinforced foams using this CNMs/CNCs hybrids, taking advantage of the possibility of using the CNCs to stabilize oil-in-water Pickering emulsions [43].

The utilization of CNFs for the dispersion of 1D CNMs has been described in different research works [8,40,50,55,60,61]. Koga et al. published the first study about the combination of CNFs, prepared by TEMPO oxidation, and SWCNTs [40]. They successfully fabricated transparent films from these dispersions, exhibiting electrical conductivity and robust mechanical properties. However, it is worth mentioning that they previously oxidized and cleaned the SWCNTs to achieve a suspension with very low SWCNT concentrations prior to the combination with CNFs, which were also highly oxidized [40]. Such a procedure might be deemed neither sustainable nor economically feasible at an industrial scale, casting doubt on whether CNFs are really stabilizing the CNMs, prompting the exploration of alternative methods. Hamedi et al. proposed the direct use of carboxymethylated CNFs to stabilize non-purified SWCNTs in water, achieving a dispersion limit above the 40 wt% [60]. They demonstrated excellent colloidal stability and applied these dispersions in different formats, including nanopapers, aerogels, and composites, all exhibiting high mechanical resistance and conductivity, validating the potential of CNFs to transfer their properties to functional materials [60]. Subsequently, they endeavored to comprehend the dispersing mechanism of CNFs with different CNTs and other CNMs [50]. They highlighted the importance of electrostatic stabilization due to the association between nanocellulose and the sp^2^ carbon surface to prevent agglomeration, which was confirmed using atomic force microscopy to follow the interaction between CNFs and graphene nanosheets. This noncovalent interaction has also been proposed by other researchers for CNCs as the reason behind the dispersing property [42]. Subsequent research works combining CNFs with CNTs show the potential application of this approach, making the hybrids suitable for 3D-printed electronics [55,61] or robust and flexible electromagnetic shields [8].

We presented a comparative study on both allomorphs of CNCs [9], prepared in a one-pot acid hydrolysis, for the dispersion of SWCNTs in water. TEM images revealed the different wrapping of the CNCs around the SWCNTs: type I CNCs were adhered parallel to the nanotube axis, while type II CNCs formed an entangled network around the nanotubes. The allomorph selection affects not only the amount of stabilized SWCNTs, but also their interaction with cells, as was tested using the Caco-2 cell line in both healthy and cancer-like behaviors. The type I-based hybrids were innocuous against this cell line, as it happened with both CNC types alone, but the type II-based hybrid presented a cytotoxic effect only against cancer cells. This selective activity was corroborated after using functionalized SWCNTs finding an even higher effect using fluorescein and folic acid as attached moieties, which could allow their use as a colon cancer theranostic agent, both as a drug and as a tool for diagnosis [44]. In parallel, we carried out another comparative study between the two hybrids for the development of electrochemical sensors of small metabolites and complex glycoproteins [45]. The type II CNCs/SWCNTs hybrid revealed a 20 times higher sensitivity in specific analytes of clinical interest, remarking again the critical role played by the selection of the cellulose allomorph [45].

The importance of choosing the appropriate CNCs allomorph based on the targeted CNM and its intended application have also been demonstrated in a recent publication, being the only one known to date to employ any cellulose nanomaterial to stabilize carbon nanofibers in water [38]. In this study, both CNCs allomorphs were used to prepare aqueous dispersions of such carbon nanofibers, with type II CNCs exhibiting exceedingly higher affinity for this 1D CNM as compared to type I CNCs, and these type II CNCs were able to stabilize carbon nanofiber concentrations up to 0.7 g/L with good colloidal stability. These dispersions, referred to as inks, were subsequently utilized to fabricate conductive textiles through the dip coating of cotton fabrics, showcasing promising thermoelectric properties. Remarkably, the characteristic n-type thermoelectric behavior of those specific carbon nanofibers was preserved even after integration into woven cotton and after thorough washing, reasserting the potential of this approach for powering wearable devices. Moreover, the enhanced affinity observed between the inks and the textile, not achieved using SDBS as dispersant of the carbon nanofibers [65], suggests that CNCs serve not only as dispersant agents but also as binders between the carbon nanofibers and the woven cotton.

The use of nanocellulose as a stabilizer has also been explored with other types of CNMs, but generally as a method aiming to prevent agglomeration or quenching rather than for direct stabilization in water [66]. An example of 0D CNMs are graphene quantum dots or carbon dots (CDs), fluorescent NPs with a diameter of only a few nanometers [66]. These nanomaterials have been combined with nanocellulose to fabricate composites with diverse applications, such as light-emitting diodes or fluorescent hydrogels [66]. However, it is important to mention that in these combinations, nanocellulose acts as a support rather than a colloidal stabilizer for the CDs, owing to its inherent stability (arguably deemed as solubility) of most CDs in water. It is important to note here that this issue has been observed in other works with CNMs, but the use of cellulose nanomaterials should reduce the use of auxiliary additives or chemical modifications for the full exploitation of the dispersion potential of CNCs and CNFs while also trying to obtain synergetic interactions.

Two-dimensional CNMs, such as graphene or graphene oxide (GO), exhibit extraordinary properties, including high mechanical strength, excellent electrical conductivity, and a large surface area, making them highly attractive for a wide range of applications such as electronics, energy storage, and biomedical devices. However, their tendency to aggregate in water due to strong π-π interactions and van der Waals forces poses a significant challenge for their end use. For this reason, there are diverse examples of combining 2D CNMs with nanocellulose materials [22,46,47,54,63]. For instance, TEMPO-oxidized CNFs have been employed to prevent the agglomeration of exfoliated graphene, resulting in dispersions of up to 1 g/L of graphene that could be transferred to nanopapers with a moisture-responsive behavior [22]. Previously, Yan et al. [63] proposed the preparation of a solution of CNFs and crumpled graphene for the development of flexible piezoresistive nanopapers, although there is no evidence in this work that the CNFs in stabilizing the graphene material. Some years later, a similar approach was published that demonstrated without a doubt the role of CNFs for stabilizing the graphene in water before the filtration to prepare the conductive nanopaper [54]. In the case of GO, nanocellulose again acts more as a support or additive rather than stabilizing the GO, as it already displays affinity for water. For example, Chen et al. [46] utilized CNCs to control the viscosity of the mixture prior to spinning with a view to obtaining fibers, which were then reduced to obtain reduced GO (rGO) composite fibers. Another noteworthy example is the combination of GO, MWCNTs, and CNCs that we proposed in a previous work [47]. Here, CNCs act as the dispersant of the MWCNTs but also as a support to prepare conductive hydrogels through hydrothermal treatment, taking advantage of the gelation properties of nanocellulose dispersions in hydrothermal conditions [47].

## 4. Other Nanomaterials Dispersed via Nanocellulose

**Figure 5:**
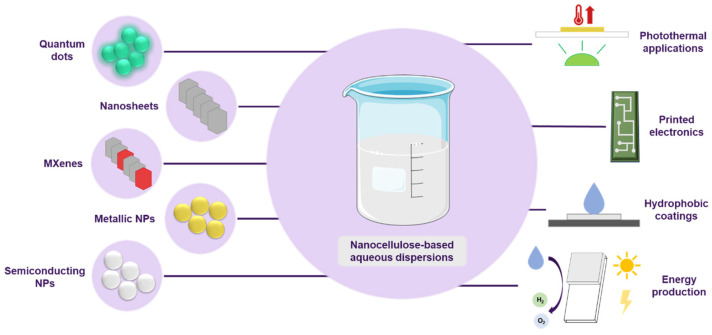
General scheme of the stabilization of different types of nanomaterials by nanocellulose in water and different areas of applications.

The combination of nanocellulose with other nanomaterials and nanostructures is a well-established topic in the scientific community, with the general idea of employing it as a structural support, additive, or template [67,68]. Nevertheless, there are few reports in which nanocellulose is directly utilized to stabilize the dispersion of these nanomaterials in water. As it occurs with CNMs, the majority of reports involving the stabilization of non-carbon-based nanomaterials in aqueous media by means of nanocellulose usually involves: (i) the chemical modification of the nanocellulose; (ii) the chemical modification of the target NPs; and/or (iii) the presence of other binders or stabilizers. However, dispersing nanomaterials in aqueous media using nanocellulose is of special interest not only for the greenness of the process, but also for the expanded range of applications in which resulting novel hybrids can be potentially employed, for which nanocellulose could offer, amongst many other things, biocompatibility and colloidal stability (Figure 5, Table 1).

The preparation of aqueous dispersions of various inorganic 2D materials in combination with nanocellulose has been addressed recently in a few publications [56,57,62]. One example refers to the use of TEMPO-oxidized CNFs for the development of stable MXene inks [56]. This required a precise control of the CNF oxidation degree for tuning the rheological properties of the as-prepared inks, which were further processed into supercapacitors by means of 3D printing technologies. Other works involving aqueous dispersions of boron nitride nanosheets (BNNS) also require the use of TEMPO-oxidized CNFs for their stabilization [57]. In this case, the great water dispersibility of the BNNS/CNF hybrids led to a substantial increase in the homogeneity and mechanical properties of the resulting films. Another example regarding aqueous dispersions of BNNS and carboxymethylated CNFs is more focused on the role of such cellulose nanostructures in the system [62]. The authors proposed that CNFs actually fulfill several roles: they serve not only as an exfoliation assistant for the BNNS but also as a BNNS stabilizer and reinforcing agent for the final nanocomposite film [62].

Regarding 0D nanomaterials such as quantum dots, the dispersion of CdS dots in aqueous media has been reported by means of TEMPO-oxidized CNFs [58]. However, CNFs were used in combination with SDBS to enhance their dispersion potential and as a requirement for application in printed electronics. There are also different examples in which nanocellulose materials are used as an aqueous stabilizer of 0D metallic NPs, normally acting as a template during their synthesis [48,51,52,53]. For instance, it has been reported that TEMPO-oxidized CNCs grafted with amino groups were able to stabilize Ag NPs in water [51]. Furthermore, the size of Ag NPs was controlled by tuning the surface chemistry of CNCs, resulting in hybrid dispersions with different stabilities and morphologies. In a similar work, two different nanocellulose materials were employed: TEMPO-oxidized CNCs and carboxymethylated CNFs for the synthesis and stabilization of Ag NPs [52]. It was demonstrated that both nanostructures play two main roles: as a template for the Ag NPs synthesis and as an ink stabilizer for printed electronics. More specifically, the ink formulation required the presence of additives and a mixture of water and isopropyl alcohol for colloidal stabilization and subsequent screen-printing processing. Au NPs have also been stabilized in water using CNCs, allowing the tuning of the mixture viscosity and their use as novel photothermal nanomaterials [53]. It is important to note that the presence of polyvinylpyrrolidone was required at the ink for increasing the compatibility between Au NPs and CNCs. Moreover, CNCs have been employed for the synthesis and stabilization of zero-valent iron NPs (nZVI) in aqueous media [48]. Several proportions of CNCs/nZVI were analyzed, finding an optimal ratio at 8/1 that yields long-term colloidal stabilization, which can be potentially employed for water remediation [48].

Reports involving the dispersion of 0D semiconducting oxide NPs in water are highly elusive to date, probably due to their challenging intrinsic water insolubility. In this sense, the successful stabilization of insoluble SiO_2_ NPs in water has been achieved by means of CNFs [64]. An increased stability of the as-prepared dispersions was obtained through the addition of methyltrimethoxysilane in the mixture, which acted as a bridge to connect CNFs by covalent bonding and SiO_2_ NPs through hydrogen bonding. Further processing of these dispersions led to superhydrophobic coatings without requiring the use of organic solvents. Another semiconducting oxide, such as TiO_2_ NPs, can be stabilized in aqueous media using TEMPO-oxidized CNFs through oil/water (O/W) emulsions [59]. As such, the stabilization of TiO_2_ NPs in the form of O/W suspensions generated a strong network between CNFs, which promoted a high viscosity as well as protection and confinement for both TiO_2_ NPs and oil droplets. Nevertheless, the former two examples still seem not to exploit the full potential of the ability of cellulose nanomaterials for NPs dispersion and stabilization, since different chemical modifications and/or adjuvants are eventually required. Recently, we proposed a novel processing of TiO_2_ NPs without requiring the oxidation of nanocellulose nor the presence of additional stabilizers [49]. Type II CNCs, prepared through one-pot acid hydrolysis with H_2_SO_4_, were utilized since this process renders nanocrystals richer in sulfate ester groups [9]. These CNCs allow the fabrication of stable waterborne TiO_2_/CNC inks even at neutral pH, which is very challenging for TiO_2_. The colloidal stability of such inks can be further enhanced just by a slight increase in the pH, using ammonium hydroxide, but with no other auxiliary adjuvant nor chemical functionalization. Furthermore, the aqueous nature of the as-prepared inks allowed their transfer into photoactive TiO_2_ films by spray-coating, being tested as photoanodes for photoelectrochemical water splitting [49]. These produced photoelectrodes demonstrated enhanced performance compared to benchmark TiO_2_ electrodes, following a greener path [49].

## 5. Conclusions and Future Outlook

This mini-review highlights the significant potential of nanocellulose (CNCs and CNFs) as a sustainable and versatile dispersing agent for various nanomaterials in water. By overcoming the limitations of conventional dispersing methods, nanocellulose provides a greener alternative that avoids harmful solvents and reduces the need for chemical modifications of the nanomaterials. Nanocellulose, derived from renewable biomass sources, displays remarkable colloidal properties in water, good mechanical properties, and biocompatibility, making it an excellent green alternative to conventional dispersion tools. Nanocellulose can effectively disperse carbon nanomaterials such as carbon nanotubes, carbon nanofibers, or graphene in water. This ability opens up applications in electronics, sensors, and energy storage while preserving the exceptional properties of CNMs in films or materials, using a greener approach, and avoiding the modification of the CNMs. The selection of the appropriate nanocellulose allomorph and type is crucial for optimal dispersion performance, depending on the specific CNM and desired application.

Furthermore, nanocellulose shows promise for dispersing challenging materials like semiconducting oxide nanoparticles, such as SiO_2_ and TiO_2_, or metallic nanoparticles. Achieving such a goal has traditionally been addressed through chemical modification of the employed nanocellulose and/or the presence of supporting stabilizers. The combination of these materials could lead to unforeseen beneficial interactions and properties, which could include selective behaviors at the chemical or biological level or even improved biocompatibility or photoelectrocatalytic activity. Further research is needed to refine the dispersion process for various nanomaterial-nanocellulose combinations. This includes optimizing factors like sonication time, nanocellulose surface chemistry, and solvent selection to achieve maximum stability and functionality. Exploring the potential of nanocellulose for dispersing a wider range of nanomaterials holds significant promise for developing novel functional hybrids. Moreover, a deeper understanding of the specific interaction mechanisms between nanocellulose and different nanomaterials is crucial for designing more targeted and efficient dispersion strategies. Reducing the number of dispersion components and steps has important implications for processing scalability, economic viability, and sustainability. Our own findings and the limited amount of the literature reported under such principles demonstrate the efficacy of this approach, offering not only colloidal stability and dispersion control but also enhanced biocompatibility stemming from the use of nanocellulose. The resulting nanohybrids could have a significant impact on a variety of applications, such as sensing, health, or energy. Further research in this area holds promise for developing more efficient methods for utilizing chemically unmodified nanocellulose and expanding its use to many other applications and nanomaterials.

## Figures and Tables

**Table 1:** Summary of the different works using nanocellulose for dispersing nanomaterials in water.

Type of Nanocellulose	Synthesis Method	Dispersed Nanomaterial	References
CNCs	Acid hydrolysis	SWCNTs	[9,39,41,42,43,44,45]
		MWCNTs	[42,43]
		Carbon nanofibers	[38]
		rGO	[46]
		GO and MWCNTs	[47]
		nZVI	[48]
		TiO_2_ NPs	[49]
	TEMPO Oxidation + Acid hydrolysis	SWCNTs	[50]
		MWCNTs	
		rGO	
	TEMPO oxidation	Ag NPs	[51,52]
	Transition metal catalyzed oxidation	Au NPs	[53]
CNFs	TEMPO oxidation	Graphene	[22]
		Graphene nanosheets	[54]
		SWCNTs	[40,50,55]
		MWCNTs	[8,50]
		rGO	[50]
		MXene	[56]
		BNNS	[57]
		CdS dots	[58]
		TiO_2_ NPs	[59]
	Acid treatment + mechanical disintegration	SWCNTs	[60]
	Carboxymethylation	SWCNTs	[61]
		BNNS	[62]
		Ag NPs	[52]
	Mechanical disintegration	Crumpled graphene	[63]
	Unavailable information	SiO_2_ NPs	[64]
